# Methodology for Real-Time Hydroplaning Risk Estimation Using an Intelligent Tire System: An Analytical Approach

**DOI:** 10.3390/s25237299

**Published:** 2025-11-30

**Authors:** Alexandru Vilsan, Corina Sandu, Gabriel Anghelache, Jeffrey Warfford

**Affiliations:** 1 Mechanical Engineering Department, Virginia Tech, Blacksburg, VA 24061, USA; csandu@vt.edu (C.S.); jwarfford@vt.edu (J.W.); 2 Automotive Engineering Department, National University of Science and Technology POLITEHNICA Bucharest, Splaiul Independenței, 313, Sector 6, RO-060042 Bucharest, Romania; gabriel.anghelache@upb.ro

**Keywords:** intelligent tires, hydroplaning, vehicle dynamics, analytical modeling, water film depth

## Abstract

This study presents a real-time capable methodology for quantifying the hydroplaning risk of a passenger car tire using data from an intelligent tire system. An analytical water lift force formulation is applied to convert measured peak lift force values into longitudinal water velocity. Based on the water velocity and groove dimensions, the intake flow rate that the tire must evacuate is estimated. Hydroplaning risk is then defined as the ratio between the intake flow rate and the maximum flow capacity of the tire before total hydroplaning occurs. Experimental investigations under real-world conditions were carried out at 45 mph and 65 mph, yielding average hydroplaning risk values of 12.6% and 21.3%. The proposed model was validated by performing hydroplaning tests under a controlled water depth of 1 mm at Michelin Laurens Proving Grounds. The hydroplaning risk values computed by the intelligent tire system were compared with reference data from the literature obtained under similar test conditions. Additionally, the critical hydroplaning speed of the test tire was estimated and compared against predictions from established numerical models, such as those proposed by Gengenbach and Spitzhüttl. The methodology is confirmed as a reliable algorithm for real-time hydroplaning risk monitoring with the potential to improve vehicle safety.

## 1. Introduction

Tire hydroplaning represents a complex phenomenon influenced by several parameters related to the tire state, such as tread depth, inflation pressure, vertical load, longitudinal speed and the road state, including water film depth and road macro-texture [1]. The risk of tire hydroplaning may be defined as the proximity of a tire to losing contact with the road surface and transitioning to complete rolling on the water layer [2]. At present, no comprehensive model exists that captures all of the relevant parameters influencing hydroplaning risk. Furthermore, even if such a model were to be developed, a significant challenge would remain in obtaining accurate, real-time measurements of these parameters to enable reliable risk estimation. This study addresses the challenge by developing a real-time hydroplaning risk assessment methodology, leveraging data acquired based on an in-house-developed intelligent tire system.

An extensive literature review on tire hydroplaning, with a focus on real-time estimation methodologies and numerical modeling for both partial and total hydroplaning phenomena, was conducted by our research group at Virginia Tech [3]. One major research direction in hydroplaning risk assessment is the development of intelligent tire systems, which integrate embedded sensors to monitor tire–road interactions in real time and provide data for improving vehicle safety and control. Comprehensive surveys were presented by Taheri [4] and Yang [5]. Tuononen [6] proposed the first intelligent tire concept for hydroplaning risk estimation, utilizing an optical sensor to identify shifts in peak vertical carcass deflection caused by the water wedge effect. On the next iteration of the intelligent tire design, Tuononen [7] replaced the optical sensor with three accelerometers mounted on the tire inner liner, aiming at detecting the threshold between partial and total hydroplaning based on longitudinal and lateral acceleration signals. Using a similar instrumentation methodology, Cheli [8] and D’Alessandro [9] were able to calculate a hydroplaning index from the radial acceleration signal measured by an MEMS accelerometer mounted on the tire inner liner. The hydrodynamic lift force and the water wedge effect modify the radial acceleration signature, producing delayed entry, signal oscillations, and a more pronounced minimum acceleration at patch exit. Hartman [10] proposes a real-time hydroplaning risk assessment system that combines external and internal sensing technologies. Road conditions are classified using a surround-view camera with computer vision and machine learning, while an intelligent tire equipped with an accelerometer detects the hydroplaning signature from radial acceleration. Data fusion enables an accurate estimation of the critical hydroplaning speed for the instrumented tire. Another research direction involves assessing hydroplaning risk using sensors already available in modern vehicles, with notable contributions from Fichtinger [11] and Blandina [1].

Model-based approaches have also been pursued. Montini [12] extended the MF-Tyre (Magic Formula) model by introducing scaling factors to represent water effects on cornering stiffness, relaxation length, friction, and motion resistance, calibrated against experimental data and linked to the volumetric drainage capacity of the tire. An ADAS (Advanced Driver-Assistance Systems) control strategy was demonstrated on a 14-DOF vehicle model, reducing speed below the critical threshold via rear-axle braking and stabilizing yaw through the cleaning effect.

In line with this research direction, this work underscores the significance of water evacuation capacity on critical hydroplaning speed, emphasizing recent analyses of groove flow mechanics in the contact patch region. Cabut [13] related the hydroplaning risk to the ability of the tire tread to evacuate water. Therefore, the research effort focused on better understanding the water flow inside the tread groove and identifying the key parameters that influence this physical phenomenon using an optical method based on Particle Image Velocimetry. The flow inside the longitudinal grooves reveals elongated white filaments, indicating air bubbles or cavities. Flow distortions can be observed, particularly in larger longitudinal grooves, displaying periodic behavior aligned with the spacing between adjacent transverse grooves. Within the contact patch area, the deformable solid walls of the groove (side walls and upper wall) are essentially stationary and the flow inside the groove approximates a channel flow with no-slip boundaries. The authors also emphasized that a significant mechanism affecting water flow in the tread grooves is vortex formation. This effect occurs near the entry of the contact patch area, where the load exerted by the tire on the layer of water causes a squeezing action, which can lead to the creation of two vortices with transverse water injection into the lower part of the groove.

Todoroff [14] and Veith [15] demonstrated in their research the influence of hydroplaning on the braking performance of both new and worn tires. Todoroff [14] introduced the concept of “hydro efficiency” to assess the tire sensitivity to hydroplaning, while Veith [15] developed the “water discharge capacity” parameter to evaluate the tire water evacuation capability. Both studies revealed that worn tires exhibit a significant reduction in braking capacity at higher longitudinal speeds, attributed to the onset of partial hydroplaning. These findings underscore the importance of developing real-time methodologies to quantify hydroplaning risk, not only to understand the proximity of the tire to complete loss of road contact, but also to enable a more accurate assessment of a vehicle’s braking performance. Lower [16] developed a test procedure for measuring the fluid pressure between the tire and the wet pavement using an inner drum test bench equipped with a piezoelectric pressure sensor. The results revealed a sharp increase in pressure as the sensor entered the tire contact patch, followed by a decline toward zero, eventually reaching a negative pressure region at the rear of the footprint where the tread lifts off the pavement. (A similar trend was observed in the present experimental setup, where the force sensor was embedded in the tire groove rather than on the road surface.) In his research, Wies [17] examined the influence of tread pattern voids on hydroplaning by applying existing hydrodynamic pressure models. A key finding relevant to the present study is the mechanism by which water floods the tread grooves. The interaction between the tread block and the wet pavement was analyzed using the Bathelt [18] rib sinkage model, which solves the Navier–Stokes equations analytically for an infinitely long pattern rib penetrating the water layer to reach the road surface. As the tread blocks contact the wet pavement, the water film is partly squeezed into the grooves and partly expelled to the edges of the contact patch. Once the grooves are flooded, the tire effectively behaves like a smooth tire. Even more importantly, the research demonstrates that groove flooding can occur even under hydroplaning conditions where the water film thickness is significantly less than the tread depth. Sinnamon [19] detailed how the squeeze-film phenomenon and water accumulation in the tread grooves are influenced by the contact patch length and the contact patch pressure distribution. For example, in the case of shallow hydroplaning conditions, the portion of the contact patch that becomes flooded with water is inversely proportional to the contact patch length and the contact patch pressure. If the inflation pressure is increased, the contact pressure increases and the contact patch length decreases. In this scenario, the net effect of the inflation pressure on the flooding of the contact patch remains small. In the case of deep hydroplaning conditions (consistent with the majority of literature on hydroplaning, where the water depth has high values above 7–8 mm), the additional hydrodynamic pressure tends to slow down the squeeze-film phenomenon, and the inflation pressure has a much greater effect on the overall hydroplaning phenomenon [19]. Consistent with the mechanism described above, an increase in tire vertical load will generate an increase in contact patch length and contact patch pressure. This will improve the tire’s overall hydroplaning behavior. Tire temperature is not specifically included in hydroplaning studies, although the expected effects on hydroplaning behavior are anticipated to follow the same mechanisms related to contact patch length and contact patch pressure distribution.

Current research on hydroplaning primarily focuses on deep water conditions where a water wedge forms in front of the tire contact patch. Most of the sensing and modeling approaches rely on this mechanism to estimate the risk of hydroplaning. As indicated in our previous research [20], the developed intelligent tire system and the proposed methodology aim to estimate the hydroplaning risk also in shallow water conditions where the water wedge effect is not always present. In this research, the hydroplaning risk is estimated based on direct measurement of the water lift force inside the tread groove and provides real-time information about the onset of partial hydroplaning. This approach does not depend on the formation of the water wedge and can therefore offer reliable hydroplaning risk estimation across both shallow and deep-water conditions.

## 2. Methodology

The following section shows the details for the data measurement process, including the components of the measurement system and the road conditions. Additionally, it presents the key steps for estimating in real-time the tire hydroplaning risk.

### 2.1. Data Acquisition Process

**Figure 1:**
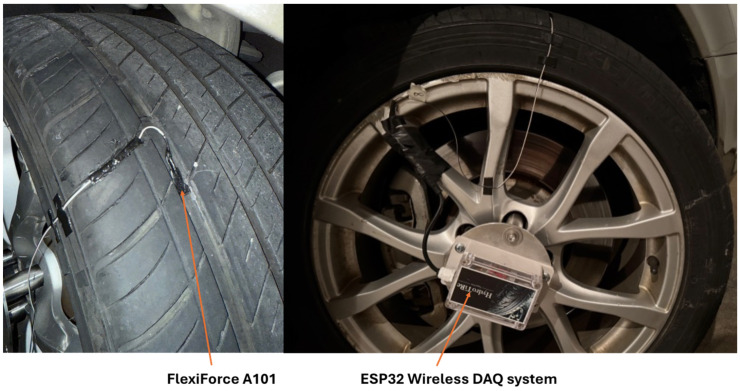
Intelligent tire for measuring hydrodynamic lift force.

An intelligent tire system has been developed by our research group at the Terramechanics, Multibody, and Vehicle Systems (TMVS) Laboratory at Virginia Tech [20]. The main components of the data acquisition system are shown in Figure 1. A 245/40 R18 all-season passenger car tire with a conventional longitudinal rib tread pattern was used for testing. The tire was not new and had been previously used for approximately 20,000 miles (32,186 km), reducing the tread depth to approximately 4.7 mm. The test vehicle is a 2009 Audi A6 equipped with an all-wheel-drive system, and the test tire was mounted on the right front wheel position.

For instrumentation, a FlexiForce A101 sensing element has been selected. According to Tekscan (333 Providence Highway Norwood, MA 02062, United States), the FlexiForce A101 is an ultra-thin and flexible sensor that functions as a pressure-sensitive variable resistor. It can measure the force between two surfaces using the piezoresistive effect, and it is durable enough to perform reliably in a wide range of operating environments. Tekscan reports typical performance characteristics, including a linearity error below 3% (full scale), repeatability error under 2.5%, hysteresis error under 4.5% (full scale), response time under 5 microseconds, and durability exceeding one million load cycles. When a vertical load is applied, the sensor’s electrical resistance decreases proportionally, allowing the applied force to be estimated by converting this resistance into a measurable voltage. For our intelligent tire system, the sensor is used to measure the water lift force generated at the interface between the tire tread and the water film. Accurate calibration between the applied vertical load and the measured voltage is essential; therefore, a detailed methodology is provided by Vilsan [20].

An ESP32 wireless data acquisition system was configured to record the sensor output at a sampling rate of 2000 Hz. The contact patch length of the instrumented tire is approximately 110 mm [20]. At the maximum tested longitudinal speed of 65 mph (104.6 km/h), the sensor is expected to measure approximately eight water lift force values while traveling through the contact patch area. Therefore, the system is able to capture the dynamic response during real-world tire–road interactions.

**Figure 2:**
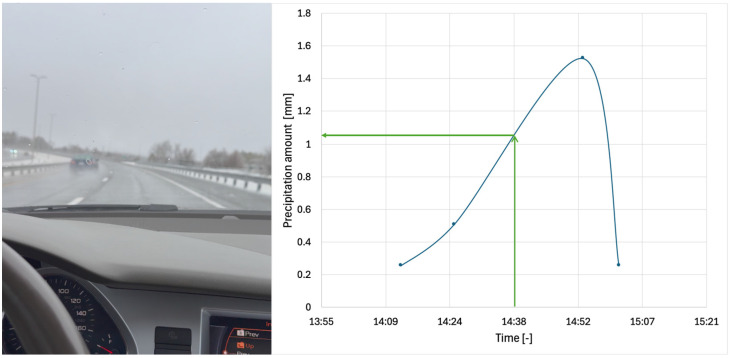
Rain conditions and measured precipitation amount.

During the experimental procedure, the tire was inflated to 280 kPa (40.6 psi) (nominal inflation pressure recommended by the vehicle manufacturer for the particular tire dimensions), and the vehicle maintained a constant speed. Figure 2 illustrates the rain conditions and the recorded precipitation amount corresponding to the date and the approximate time interval of the measurements (on a portion of US 460, in Blacksburg, Virginia, 12 February 2025 at 14:38). The reported values represent the change in precipitation amount during each measurement interval. Although the water film depth was not measured directly due to the complexity of the process, rainfall intensity reported by a local weather station indicated light to moderate rain during the measurement period. The next paragraph outlines a methodology for establishing a correlation between precipitation amount and water film depth on US highways.

For example, data on water film depth and precipitation amount were recorded by the Maryland Department of Transportation (MDOT) at one of their weather stations from 15 November 2022 to 15 November 2023. This data set is available for analysis through a Freedom of Information Act (FOIA) request. The weather station designated “I68 at US219” is located in Keysers Ridge, Maryland. Further details regarding the measurement capabilities of the MDOT system are presented by Vilsan [21].

**Figure 3:**
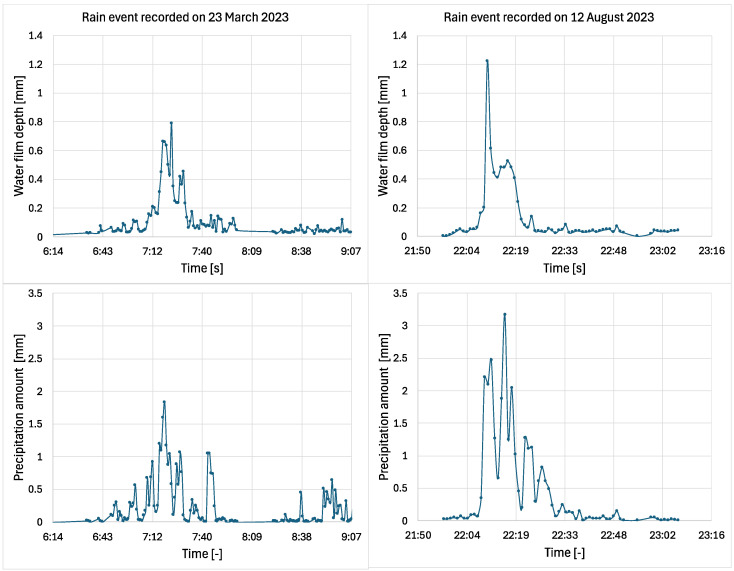
Correlation between precipitation amount and water film depth for two separate rain events on highway I68.

Even though the location where the intelligent tire data was collected differs from the MDOT measurement location, the analysis can still provide valuable and reasonably accurate insights into the variation in water film depth as a function of precipitation amount, as presented by Figure 3.

**Figure 4:**
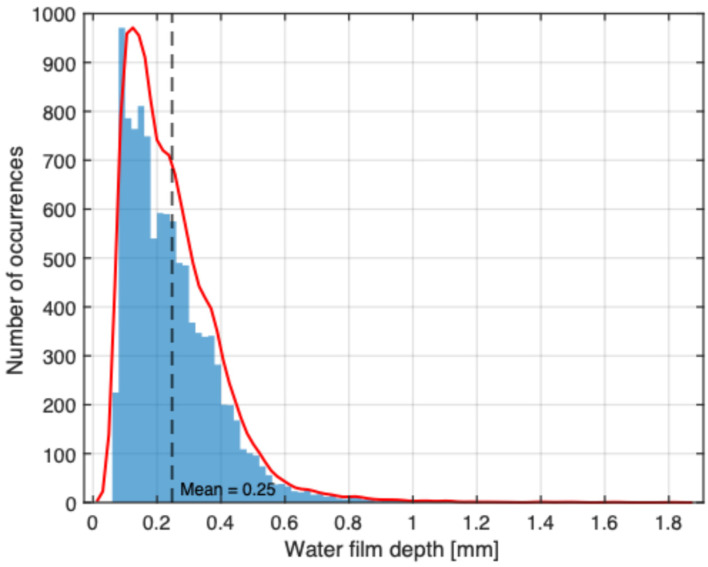
Distribution of measured water film thickness reported by MDOT.

Figure 3 shows a clear correlation between the water film depth on the road and the measured precipitation amount. Similarly to Figure 2, the precipitation values indicate the amount recorded during each measurement interval, not cumulative totals. The peak water film depth recorded during the 23 March 2023 event was 0.8 mm and the peak value for the precipitation amount was 2 mm. Similarly, during the 12 August 2023 event, the recorded water depth peak was 1.2 mm, and the precipitation amount peak was 3 mm. Furthermore, a precipitation of 1 mm corresponds to a water film depth of 0.2 to 0.3 mm. Although local factors, such as pavement slope, pavement texture, pavement drainage, and wind speed, can influence the relationship between the precipitation amount and the water film depth, the proposed method can be useful for estimating the water film depth during the measurements. Therefore, for the same precipitation amount during the experimental measurements, it is expected that the water film depth will have similar values of 0.2–0.3 mm. Figure 4 shows the frequency distribution of measured water film thickness reported by MDOT for the entire year.

The results reveal that the majority of the samples correspond to shallow water depths below 0.5 mm, with a mean value of approximately 0.25 mm. This confirms that the most probable water depth for the real-world test conditions lies between 0.2 mm and 0.3 mm. The assumption is further supported by water film depth data reported by Biesse [22]. Measurements from 587 road weather stations across Germany, collected over a period of 278 days, show that nearly 99% of the recorded water film depth values fall within the 0–1 mm range. Similarly, data collected over two years from 17 road weather stations in France indicate that more than 99% of water depth measurements also fall within this range. In addition, mobile measurements conducted over 640 km of wet roads in France confirm the same trend observed at fixed stations. Based on this evidence, Biesse concluded that water film depths exceeding 1 mm occur in less than 1% of cases and are typically associated with intense summer storms.

### 2.2. Real-Time Hydroplaning Risk Estimation Methodology

Horne [23] noted that fluid inertia effects tend to slow down the escape of water from the tire–ground contact region, facilitating the formation of a fluid film that can separate the tire tread from the pavement. When the tire reaches the critical hydroplaning speed, the hydrodynamic lift generated beneath the tire equals the portion of the vehicle’s weight carried by the tire. Any further increase in speed beyond this critical point would cause the tire to completely lift off the road surface. For the entire contact patch area, the lift force *F*_*V*,*S*_ is calculated using Equation (1).

(1) $$F_{V,S}=\frac{1}{2}C_{L,S}\;\rho \;A_{G}\;V^{2}$$

where *C*_*L*,*S*_ represents the dimensionless hydrodynamic lift coefficient, *ρ* represents the fluid mass density, *A_G_* represents the gross tire contact area (including the tread grooves) and *V* represents the tire longitudinal speed.

Martin [24] notes that the lift coefficient should vary with tire inflation pressure and water film depth, given the influence of these factors on the water groove flow. Nevertheless, Martin also highlights that, across a range of tests covering tire inflation pressures from 24 to 150 psi (165 to 1034 kPa), vehicle speeds from 45 to 120 mph (72.4 to 193.1 km/h), and vehicle loads from 925 to 22,000 lbs (419 to 9979 kg), the lift coefficient measured by Horne remained consistently at 0.7 [23].

**Figure 5:**
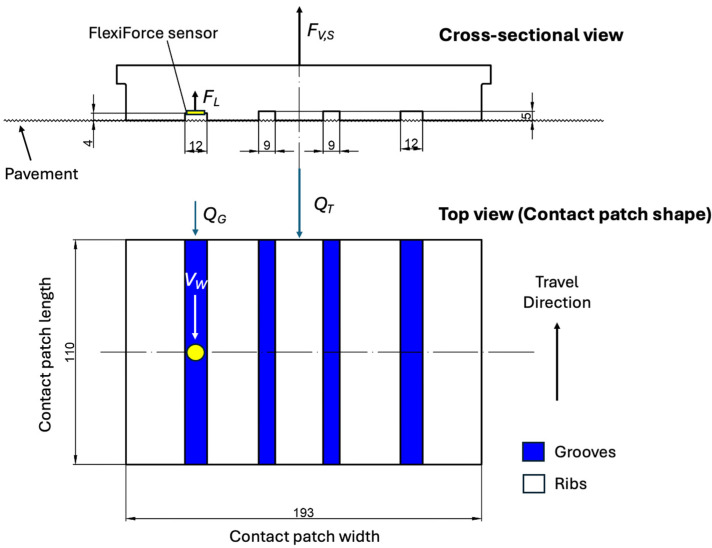
Water flows through the groove channels for the test tire.

Out of the total lift force *F_V,S_*, the intelligent tire described in the previous section will measure only a fraction, denoted as *F_L_* in Figure 5 and Equation (2).

(2) $$F_{L}=\frac{1}{2}C_{L,S}\;\rho {\;A}_{s}\;V_{w}^{2}\;$$

In Equation (2), *A_S_* represents the sensing area of the FlexiForce sensor, which is placed in one of the tread grooves, and *V_W_* represents the longitudinal speed of water within that groove.

The longitudinal speed of water through the groove *V_W_* is calculated using Equation (3).

(3) $$V_{W}=\sqrt{2\frac{F_{L}}{C_{L,S}\;\rho {\;A}_{s}}\;}$$

As previously mentioned in Section 1, Cabut [13] stated that the flow inside the groove approximates a channel flow with no-slip boundaries. Therefore, the water flow rate through the groove *Q_G_* is calculated as the product between the longitudinal speed of water and the flow cross-sectional area, which is defined by the product between groove depth *GD* and groove width *GW*, as shown in Equation (4).

(4) $$Q_{G}=\sqrt{2\frac{F_{L}}{C_{L,S}\;\rho {\;A}_{s}}\;}\;GD\;GW$$

The water flow through each of the tread grooves occurs in two stages. In the first stage, when the tread blocks contact the pavement asperities, water fills only a portion of the tread grooves corresponding to the water film depth on the road surface. In the second stage, the squeeze film mechanism helps fill the remaining tread groove voids with water [17]. This mechanism explains why groove flow occurs even when the water film depth is significantly lower than the groove depth. The total intake flow rate that the tire needs to evacuate *Q_T_* is calculated using Equation (5).

(5) $$Q_{T}=\frac{1}{0.24}\sqrt{2\frac{F_{L}}{C_{L,S}\;\rho {\;A}_{s}}\;}\;GD\;GW\;$$

It can be observed that the test case tire exhibits varying groove depths due to asymmetrical wear, as well as different groove widths that are a result of the tread pattern design developed by the tire manufacturer. The sum of the groove cross-sectional areas is 198 mm^2^, while the cross-sectional area of the groove where the FlexiForce sensor is positioned is 48 mm^2^. Therefore, as an approximation, the groove flow rate *Q_G_* is expected to be 24% of the total intake flow rate *Q_T_*, which explains the first term of Equation (5).

As mentioned in Section 1, the tire can evacuate only a certain amount of water until total hydroplaning occurs. A methodology for calculating the intake flow rate limit, known as the choke flow rate *Q_C_*, was introduced by our research group [20] based on the water flow model for locked tires proposed by Sinnamon [19].

(6) $$Q_{c}=3.53\;\sqrt{p}\;TW\;\left(1-CSR\right)\;TD$$

The choke flow rate for a locked wheel is a function of inflation pressure *p*, tread width *TW*, contact surface ratio *CSR* and average tread depth *TD*.

The hydroplaning risk *HR* is defined as the proximity of a tire to losing contact with the road surface by transitioning to complete rolling on the water layer. The hydroplaning risk has values between 0% and 100% and it is calculated as the ratio between the total intake flow rate *Q_T_* and the choke flow rate *Q_C_* as indicated in Equation (7).

(7) $$HR=1.2\;\frac{\sqrt{2\frac{F_{L}}{C_{L,S}\;\rho {\;A}_{s}}\;}\;GD\;GW}{\sqrt{p}\;TW\;\left(1-CSR\right)\;TD}\cdot 100\%$$

According to Sinnamon [19], a locked tire is expected to experience hydroplaning sooner than a rotating tire. Therefore, the results computed with Equation (7) are expected to be more conservative compared to real-world results. On one side, this approach is desirable and can serve as a safety factor in hydroplaning risk estimation. On the other side, a conservative risk estimate can trigger earlier warnings or interventions of ADASs. Such responses do not compromise safety, but they may limit driver authority more than required. The methodology presented can be leveraged to improve the overall safety of the vehicle, but maintaining a balance between safety margins and driver authority remains essential.

Table 1 presents the values of each parameter considered in applying Equation (7) to the test tire used during the measurements.

It can be observed that after all parameters of the test tire are determined, the risk of hydroplaning can be estimated in real-time with the intelligent tire system only by measuring the lift force of water in one of the tire tread grooves.

## 3. Results and Discussion

This section analyzes the lift force data measured using the intelligent tire system in the time domain and frequency domain. Furthermore, it discusses the results obtained from processing the measured data by applying the methodology for real-time hydroplaning risk estimation presented in the previous section.

### 3.1. Water Lift Force Measured in the Tread Groove

**Figure 6:**
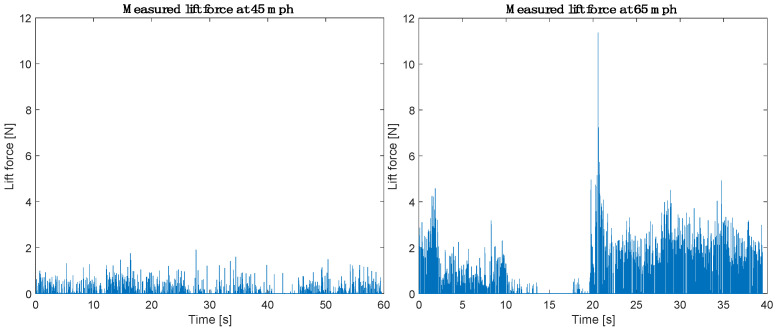
Lift force measured at 45 mph and 65 mph.

Two experiments have been performed under the same road conditions at longitudinal speeds of 45 mph (72.4 km/h) and 65 mph (104.6 km/h), as presented in Figure 6. The recordings were made two minutes apart between 14:38 and 14:40 on 12 February 2025. The water depth is assumed to be constant during both tests.

Figure 6 shows that at the lower speed of 45 mph (72.4 km/h), the lift force measurements are relatively small and fluctuate less, with peak lift force values below 2 N. At 65 mph (104.6 km/h), the lift force signal shows significantly higher amplitudes and greater variability, with peak lift force values above 2 N. The variation in the lift force data can be attributed primarily to the differences in longitudinal speed, and not to changes in the road conditions (the effect of the passage of other vehicles was negligible, as the road was relatively empty during testing). The observed behavior is consistent with Equation (2). As the longitudinal speed increases, the speed of water traveling through the tread grooves also increases, which in turn raises the overall lift force measured by the FlexiForce sensor.

Real-world measurements are affected by the presence of electrical noise. It is important to quantify the signal-to-noise ratio *SNR* before using the lift force data for estimating the tire hydroplaning risk. *SNR* provides a good estimate of how strong an electrical signal is in comparison with the background noise. For this research, the *SNR* will be expressed in decibels (dB), according to Equation (8), where *A_s_* and *A_N_* represent the root mean square amplitudes of the signal and, respectively, of the noise [25].

(8) $$SNR=20\;\log_{10}\left(\frac{A_{S}}{A_{N}}\right)$$

**Figure 7:**
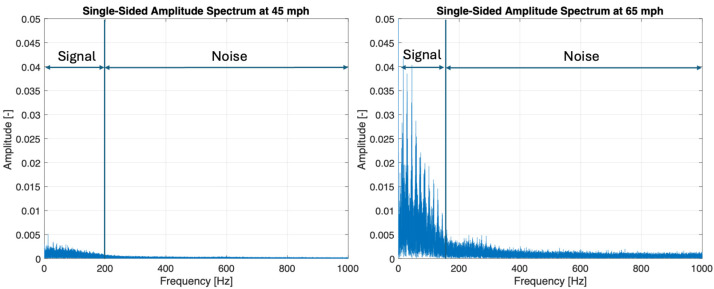
Single-sided amplitude spectrum for 45 mph and 65 mph data sets.

The time-domain data presented in Figure 6 does not reveal a clear distinction between the signal and the noise. Therefore, the data has been shifted into the frequency domain using MATLAB’s Fast Fourier Transform algorithm (MATLAB_R2024a). Figure 7 presents the single-sided amplitude spectrum for both data sets at 45 mph (72.4 km/h) and 65 mph (104.6 km/h).

Figure 7 shows that the signal components are concentrated below 200 Hz for the 45 mph (72.4 km/h) test and below 150 Hz for the 65 mph test (104.6 km/h), while the noise floor remains relatively constant and with lower amplitudes beyond the mentioned frequency ranges. The results are partially consistent with the findings of Hartmann [10], who analyzed radial acceleration signals measured by an intelligent tire system (eTIS) under partial hydroplaning conditions. In the study, a vehicle equipped with eTIS sensors was driven through a water basin (approximately 10–20 mm deep) at 60 km/h, just below the full hydroplaning threshold of 75 km/h. Spectral analysis of the radial acceleration revealed an increased spectral density at relatively low frequencies (0–200 Hz), which was observed to be distributed symmetrically across the entire contact patch area.

Applying Equation (8) for the data presented in Figure 7, the calculated *SNR* value for 45 mph (72.4 km/h) is 14.6 dB, while the *SNR* value for 65 mph (104.6 km/h) is 18.5 dB. The amplitude of the signal is approximately five times greater than the amplitude of the noise for the 45 mph (72.4 km/h) test case, and approximately eight times greater for the 65 mph (104.6 km/h) test case. Vaahteranoksa [26] noted that a good enough *SNR* value for video recording is in the range between 15 and 17.5 dB. While clear thresholds for the *SNR* values are not available for the proposed measurement system, it can be concluded that the signal is significantly stronger than the noise. Therefore, the measured data is suitable for further processing to estimate the tire hydroplaning risk.

### 3.2. Hydroplaning Risk Estimation

**Figure 8:**
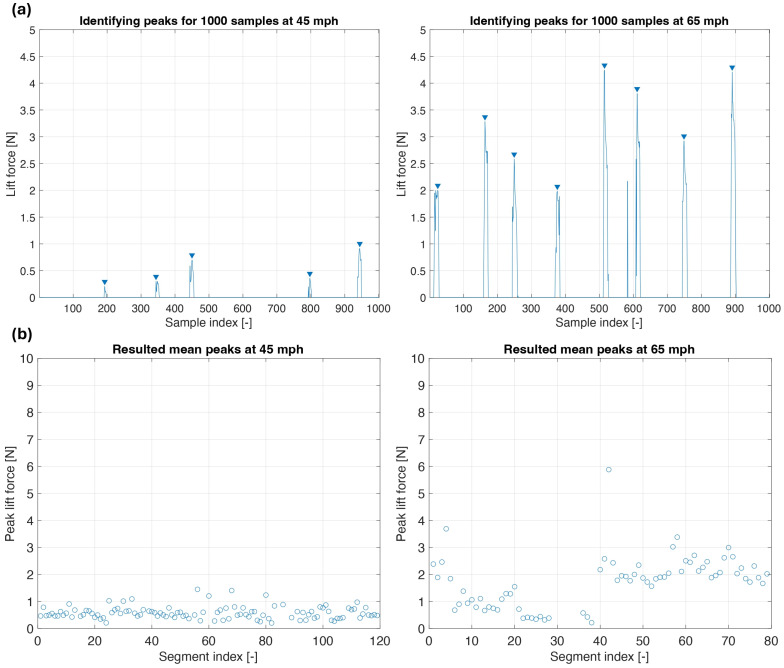
(**a**) Identifying peaks for the measured lift force; (**b**) average lift force peak values at 45 mph and 65 mph.

The first step for estimating the tire hydroplaning risk is to separate the lift force data into 0.5 s segments (1000 samples at a 2000 Hz sampling rate). Figure 8a illustrates how the lift force evolves as the FlexiForce sensor enters and exits the contact patch area during one such segment. As expected, at higher longitudinal speeds, more pressure peaks are detected within the same number of samples because the sensor enters the contact patch more frequently during the same time interval. Within each 0.5 s segment, the peak lift force values are identified using a peak detection algorithm in MATLAB. An average of the peak values is then computed for each segment, resulting in a representative peak lift force value per 1000 sample window. Figure 8b presents the average peak lift force values computed for all segments across the entire data sets of 45 mph (72.4 km/h) and 65 mph (104.6 km/h). The 45 mph (72.4 km/h) data set indicates that the water flow is stable with limited turbulent effects. The 65 mph (104.6 km/h) data set presents significantly higher peak lift force values and, also, higher data variability.

**Figure 9:**
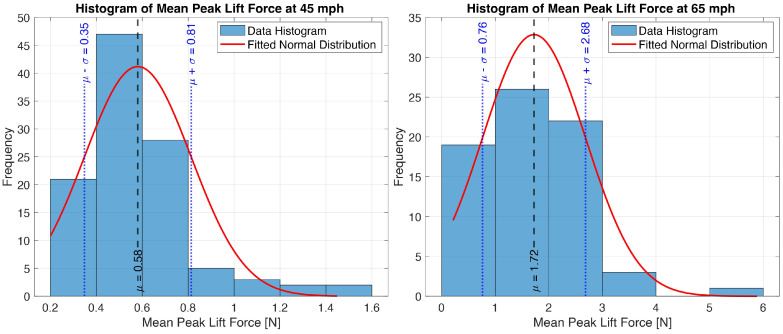
Statistical analysis for the average peak force data.

Using Particle Image Velocimetry, Cabut [13] observed the formation of air bubbles and cavities in the longitudinal grooves, indicating the presence of a two-phase flow. The air bubbles are disrupting the uniformity of the water flow, causing variation in the measured lift force. Furthermore, turbulent flow effects within the grooves are increasing with the longitudinal speed. Additionally, local variations in water film depth and pavement drainage capabilities can also contribute to the variability of the lift force measurements. Figure 9 presents a statistical analysis of the data from Figure 8b.

The histograms in Figure 9 show the distribution of the average peak lift force values at both 45 mph (72.4 km/h) and 65 mph (104.6 km/h). The red line represents the normal distribution fitted for the measured data. The solid red line represents the normal distribution fitted to the measured data. The vertical dashed black line indicates the mean of the distribution, while the blue lines represent one standard deviation above and below the mean. For the 45 mph (72.4 km/h) test case, most of the measured lift force peaks are clustered around the mean value. In contrast, at 65 mph (104.6 km/h), the histogram shows both a larger standard deviation and a mean with a significantly higher value. The larger standard deviation indicates that while most lift force values remain moderate, there is a higher probability of encountering larger force peaks, potentially increasing the hydroplaning risk at higher longitudinal speeds.

**Figure 10:**
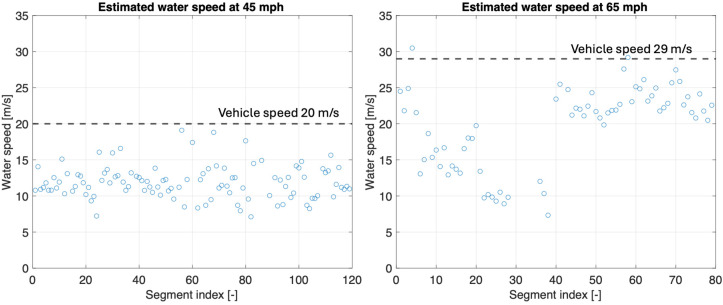
Estimated water speed at 45 mph and 65 mph.

The second step in estimating the hydroplaning risk of the tire is to determine the longitudinal speed of the water in the groove channel. The average peak force data presented in Figure 8b is used in Equation (3) to estimate this water speed. Figure 10 shows the results obtained for both the 45 mph (72.4 km/h) and 65 mph cases (104.6 km/h).

The estimated water speeds in the tread groove are consistently lower than the vehicle speed. For the 45 mph test case (20 m/s vehicle speed), the estimated water speeds are in the range between 10 and 15 m/s. For the 65 mph test case (29 m/s vehicle speed), the estimated water speeds are higher, between 10 and 26 m/s, but still below the vehicle speed. These results align with the findings reported by Cabut [13], who measured the instantaneous water velocity within the longitudinal tread grooves of a passenger car tire. The researcher observed that the local water speed can be significantly lower than the vehicle speed, with absolute values as low as 10% of the vehicle speed. It is important to note that the data presented in Figure 10 represents the maximum water speed values encountered in the contact patch, calculated from the peak force values measured with the FlexiForce sensor.

**Figure 11:**
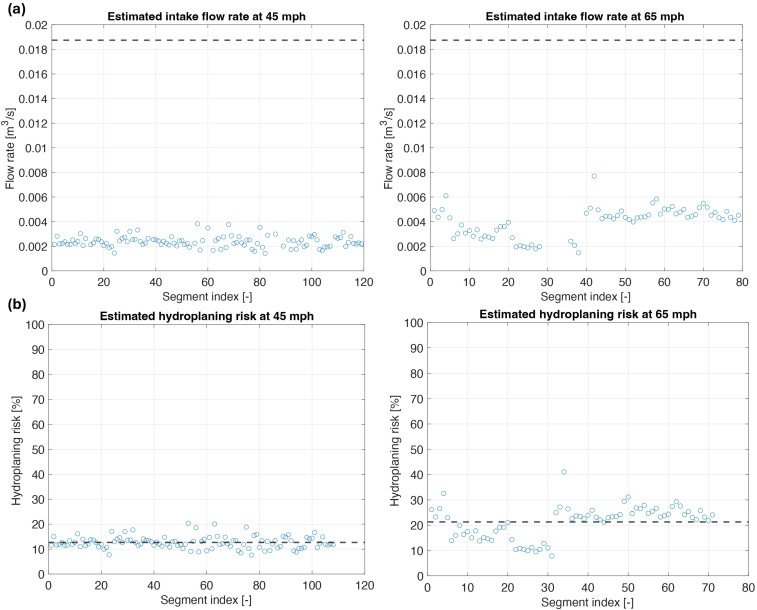
(**a**) water intake flow rate prediction; (**b**) estimated hydroplaning risk at 45 mph and 65 mph.

The next step is to estimate the total intake flow of water at the front of the tire contact patch, illustrated in Figure 11a, by applying Equation (5), which incorporates the previously estimated water speed values and the groove dimensions. The intake flow rates cluster around lower values, with no apparent peaks approaching the choke limit. This suggests that, under the tested conditions, the flow rate through the tire grooves remains well below the maximum theoretical intake, indicating that the grooves do not operate at maximum flow capacity at either speed. This is consistent with typical behavior for a tire with a good tread pattern operating under shallow water depths, where total hydroplaning is expected to occur at much higher longitudinal speeds [20,21]. According to the methodology presented in Section 2.2, the last step for computing the hydroplaning risk is to divide the total intake flow rate by the choke flow rate limit, as given by Equation (7). Figure 11b presents the hydroplaning risk data expressed as a percentage, where 0% indicates no hydroplaning risk and 100% indicates that the tire is experiencing total hydroplaning.

For most of the estimated points, the hydroplaning risk remains below 25%, with mean values of 12.6% at 45 mph (72.4 km/h) and 21.3% at 65 mph (104.6 km/h). The dashed lines indicate these average risk levels. It is important to note that the choke flow rate used for the risk calculation corresponds to a locked wheel condition. As mentioned previously, a rotating tire typically hydroplanes at higher longitudinal speeds compared to a locked tire. Therefore, the risk results presented may be slightly higher than the real-world risk levels for a rotating tire. This should be considered when interpreting the results and comparing them to actual vehicle performance on wet surfaces.

For shallow hydroplaning conditions, our research group [21] identified that the critical hydroplaning speed can be calculated using the Gengenbach model [27], per Equation (9).

(9) $$V_{p}=508\;\sqrt{\frac{Q}{B\;t\;C_{l}}}$$

The critical hydroplaning speed *V_p_* (km/h) is expressed as a function of vertical load *Q* (kg), contact patch width *B* (mm), water film thickness *t* (mm), and lift coefficient *C_l_,* which is dimensionless. The lift coefficient defined by Gengenbach does not possess the same physical interpretation as the lift coefficient introduced by Horne [23]. It represents an empirical parameter derived from curve fitting of experimental data. Gallaway [28] reported that this coefficient is strongly influenced by tread pattern design, with values ranging from 46 for slick tires to 15.5 for tires with lateral grooves. He also noted that a longitudinally grooved tread pattern corresponds to a lift coefficient of approximately 26. For an estimated water depth in the range of 0.2–0.3 mm, a measured vertical load of 550 kg, and a measured contact patch width of 193 mm (same as the tread width *TW*), the Gengenbach hydroplaning model predicts a critical hydroplaning speed in the range of 190–233 mph (307.1–376.1 km/h). This supports the finding of a low average hydroplaning risk at 45 mph (72.4 km/h) and 65 mph (104.6 km/h).

**Figure 12:**
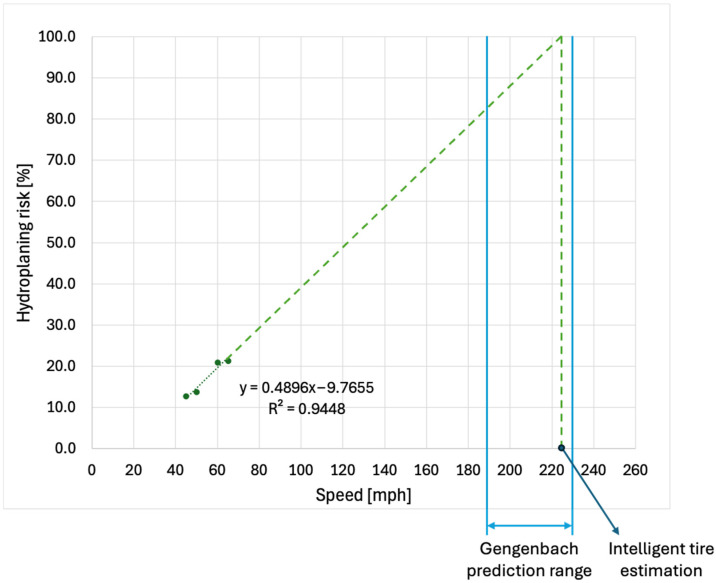
Hydroplaning risk as a function of longitudinal speed for real-world conditions.

Using the intelligent tire system, two additional average hydroplaning risk points were measured and analyzed at 50 mph (80.5 km/h) and 60 mph (96.6 km/h), following the procedure described in Section 2.2 and Section 3.2. Figure 12 shows the evolution of hydroplaning risk as a function of longitudinal speed.

The evolution of hydroplaning risk as a function of vehicle speed indicates a critical hydroplaning threshold (*HR* = 100%) at 224 mph (360.5 km/h). This value is approximately 17.4% higher than the lower limit of the Gengenbach range and only 3.9% lower than its upper limit, demonstrating a reasonable agreement between the two independent approaches. Several sources of error may account for the observed discrepancy. First, the water film depth was not directly measured, and the assumed water depth interval may have been overestimated. Second, the test tire may exhibit inferior water evacuation capability relative to the reference tire used in the Gengenbach study. In such a case, the effective lift coefficient would be higher than the assumed value of 26, which would lead to a lower critical hydroplaning speed in the Gengenbach formulation. The variation in hydroplaning risk with vehicle speed further suggests that the tire is operating under shallow hydroplaning conditions. In addition, the hydroplaning risk begins to deviate from zero at longitudinal speeds above 20 mph (32.2 km/h), indicating the onset of partial hydroplaning. This trend is in good agreement with the observations of Hermange [29], who reported that the tire begins to lose part of its contact area due to partial hydroplaning at similar longitudinal speeds.

## 4. Model Validation

**Figure 13:**
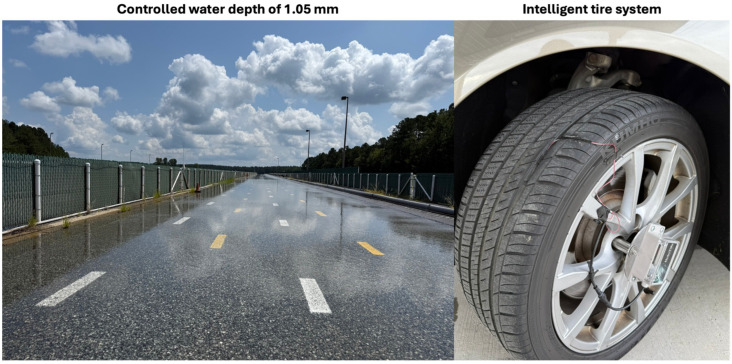
Testing conditions for validating the hydroplaning model.

This section presents the method used to validate the hydroplaning risk model developed in the previous chapters. The validation approach involves comparing the hydroplaning risk data obtained with the intelligent tire system during controlled tests at a water film depth of 1.05 mm with literature data obtained from optical observations of the tire contact patch filmed under similar conditions. The validation tests were performed at Michelin Laurens Proving Grounds in South Carolina. One of the main sources of uncertainty in the presented hydroplaning data is the estimation of the water film depth. While most standardized hydroplaning tests are performed at a water depth of approximately 8 mm, this value does not reflect typical conditions encountered in real-world driving. To address this gap, the tests in this study were specifically performed at a controlled water film depth of 1.05 mm, in order to better represent realistic wet-road scenarios. Figure 13 illustrates the testing setup and environmental conditions used during the validation experiments.

Per Michelin’s standard test protocols, the water depth was maintained approximately constant along the longitudinal direction of the test track (150 m in length), while in the lateral direction it varied for different fixed values below the maximum of 1.05 mm, according to the marked reference lines shown in Figure 13. For these experiments, the instrumented tire was mounted on the left front wheel position to ensure that the test tire encountered the desired water depth. The tests were conducted on 21 August 2025, more than six months after the initial real-world measurements on US 460. During this interval, the tires accumulated an additional 4000 miles (6437 km), and the wear-related parameters summarized in Table 1 were updated accordingly. The groove depth *GD* of the instrumented channel decreased to 3.7 mm, while the average tread depth *TD* reached 4.4 mm. The total cross-sectional area of the grooves was measured at 180.3 mm^2^, with the groove instrumented with the FlexiForce sensor having an area of 44.4 mm^2^. Based on these updated values, the groove flow rate *Q_G_* is estimated to represent 24.6% of the total intake flow rate *Q_T_*, thereby modifying the first term of Equation (5).

**Figure 14:**
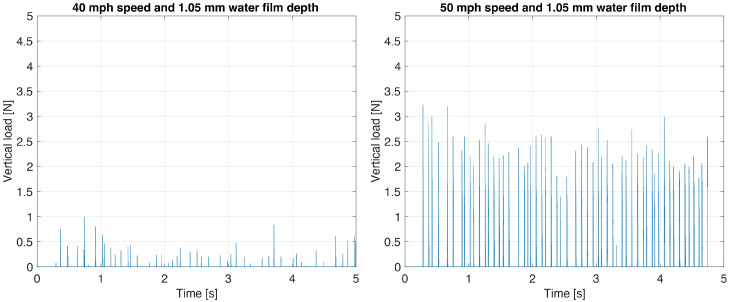
Water lift force for 40 mph and 50 mph at same water depth.

Figure 14 shows the measured lift force signal for two longitudinal speeds at 40 mph (64.4 km/h) and 50 mph (80.5 km/h), and a controlled water depth of 1.05 mm.

**Figure 15:**
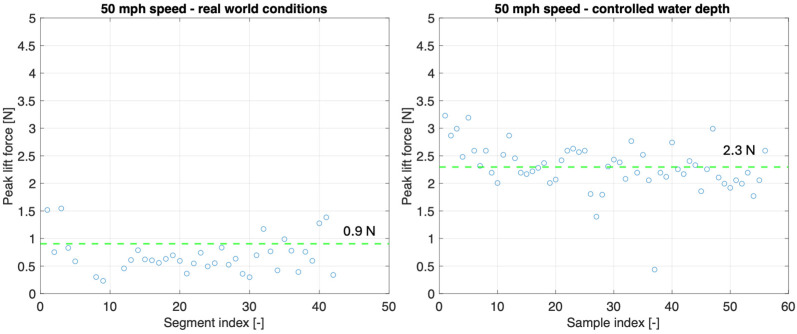
Peak water lift force at same speed but different water depth.

At a water film depth of 1.05 mm, the measured vertical load signal exhibits distinct features between the two tested longitudinal speeds. At 40 mph (64.4 km/h), the signal is characterized by very low peak amplitudes, not exceeding 0.5–1 N. By contrast, at 50 mph (80.5 km/h), the signal displays a marked increase, both in amplitude and in frequency of occurrence. The vertical load peaks regularly exceed 2.0 N, with the highest values approaching 3.5 N. These observations confirm that, for the same controlled water depth, the intelligent tire system correctly detected the expected increase in water lift force associated with a higher longitudinal speed. Figure 15 compares the measured peak water lift force at 50 mph (80.5 km/h) for the controlled water depth test conducted on 21 August 2025 and the real-world test conducted on 12 February 2025. Due to the limited amount of data available for the controlled water depth tests, the data set is no longer divided into 1000 sample segments for computing the average peak value.

At a longitudinal speed of 50 mph (64.4 km/h), the measured peak lift force shows a clear dependency on the water depth. Under controlled water depth conditions of 1.05 mm, the average peak lift force reached 2.3 N, whereas under real-world conditions, the average value was only 0.9 N. The significant reduction in the lift force values confirms that the actual water depth encountered during real-world testing was substantially lower than the water depth maintained under controlled conditions. Assuming a linear dependency of lift force on water film depth (for shallow hydroplaning conditions), the measured results suggest that the water depth in real conditions was approximately 0.4 mm, which is consistent with the estimated value reported in Section 2.1. This outcome indicates that the intelligent tire system is sensitive to water depth variations.

**Figure 16:**
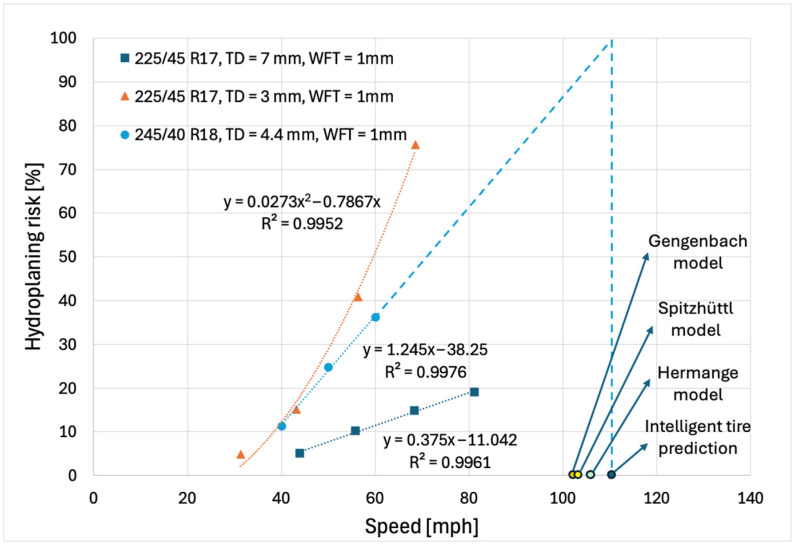
Hydroplaning risk as a function of speed for controlled test conditions (adapted from [29]).

Hermange [29] analyzed high-speed camera images that capture the contact patch behavior during partial and total hydroplaning, with the camera mounted below a transparent pavement section. To establish a reference surface area, *S0*, the tests were conducted at a constant speed of approximately 20 mph (32.2 km/h), where hydroplaning effects are minimal, and the contact patch dimensions are stable. The critical hydroplaning speed was determined as the speed at which the contact surface area, *S*, between the tire and the measurement window approaches zero. Some of the data published by Hermange [29] will be adapted to align with the hydroplaning risk data presented in the previous section. In this study, a ratio of *S/S0* of 0% indicates a 100% hydroplaning risk, while an *S/S0* of 100% corresponds to a 0% hydroplaning risk. Figure 16 shows the evolution of hydroplaning risk as a function of longitudinal speed for both the tested tire equipped with the FlexiForce sensor and the reference tires from Hermange’s study [29].

Using the intelligent tire system in controlled water depth conditions, three hydroplaning risk points were measured and analyzed at 40 mph (64.4 km/h), 50 mph (80.5 km/h), and 60 mph (96.6 km/h), following the procedure described in Section 2.2 and Section 3.2. The hydroplaning risk data points were collected at the Michelin proving ground in Clermont-Ferrand, France, under a water depth of 1 mm, using two separate tires, one with a tread depth of 7 mm and one with a tread depth of 3 mm. Following Sinnamon groove flow theory [19], the tire with a 7 mm tread depth is experiencing shallow hydroplaning conditions, where the intake flow rate varies linearly with longitudinal speed, resulting in a linear relationship between hydroplaning risk and vehicle speed. The intelligent tire used for this study also exhibits shallow hydroplaning behavior, and the hydroplaning risk varies linearly with vehicle speed. In contrast, the tire with a 3 mm tread depth is in deep hydroplaning conditions, where a water wedge forms ahead of the contact patch, causing the intake flow rate (and hydroplaning risk) to vary with longitudinal speed squared.

Several important differences should be noted between the instrumented tire and the reference tires. First, the reference tires were tested on a glass panel, which prevents water drainage into pavement asperities. Under real-world conditions, the same tires would likely exhibit lower hydroplaning risk. Second, the cross-sectional width of the test tire was larger, resulting in a wider tread. This characteristic typically reduces hydroplaning performance compared with narrower reference tires [19,27]. Third, the inflation pressure of the reference tires was lower (220 kPa) than that of the test tire (280 kPa). However, for shallow water depths, this parameter is not expected to play a significant role in hydroplaning behavior. Fourth, the vertical load applied to the reference tires was not reported, but a qualitative assessment of the test vehicle indicates that it was lower than the load applied to the test tire. A lower vertical load generally reduces hydroplaning performance. The tread designs of the test tire and the reference tire are similar; therefore, the tread pattern is unlikely to account for the observed differences.

Although the test conditions and tire specifications differ, the hydroplaning risk estimation algorithm produces results within the expected range, between those of the Hermange reference tires with tread depths of 3 mm and 7 mm. For all three cases, hydroplaning risk begins to deviate from zero at approximately 30 mph (48.3 km/h), marking the onset of partial hydroplaning. The critical hydroplaning speed predicted by the intelligent tire system (*HR* = 100%) is 111 mph (178.6 km/h), which is substantially lower than values reported under real-world conditions. This discrepancy further confirms that the effective water depth in real conditions is significantly lower than the controlled depth of 1.05 mm. Using Equation (9) with the same instrumented tire parameters and a water depth of 1.05 mm, the Gengenbach model predicts a critical hydroplaning speed of 102 mph (164.2 km/h). The relative difference between the two predictions is 8%. As in the real-world case, the Gengenbach model yields a lower critical speed compared to the proposed methodology.

Based on experimental data, Hermange [29] has introduced a new equation for calculating the critical hydroplaning speed *V_p_* (km/h) as a function of water absorption capability *γ*_1_.

(10) $$V_{p}=69\gamma_{1}+69.5\;$$

The water absorption capability is defined in the literature [19] as the ratio between the tire groove capacity *g* and the water film depth *h*. The tread groove capacity *g* is calculated based on average tread depth *TD* and contact surface ratio *CSR* according to Equation (11) [20,29].

(11) $$\gamma_{1}=\frac{g}{h}=\frac{\left(1-CSR\right)\;TD}{h}$$

For a contact surface ratio of 0.65, an average tread depth of 4.4 mm, and a water depth of 1.05 mm, the water absorption capability *γ*_1_ of the intelligent tire is 1.47. Applying the Hermange model (Equation (10)), the predicted critical hydroplaning speed is 106 mph (170.6 km/h). This value remains lower than that predicted by the proposed methodology, but the relative difference is reduced to 5%.

Spitzhüttl [30] proposed a model similar to that of Hermange for calculating the critical hydroplaning speed *V_p_* (km/h) as a function of the water absorption capability *γ*_1_ and the tire inflation pressure *P* (bar).

(12) $$V_{p}=52{\;\gamma }_{1}+16.4\;\left(P-2.2\right)+79$$

For an inflation pressure of 280 kPa (2.8 bar) and assuming the same water absorption capability of 1.47, this model predicts a critical hydroplaning speed of 103 mph (165.8 km/h). The calculated value further supports the accuracy of the intelligent tire system predictions and the proposed hydroplaning risk estimation methodology.

To improve clarity, the validation results presented above can be viewed as three complementary pathways, each supporting a different aspect of the proposed hydroplaning risk estimation method. First, the controlled experimental validation confirms that the intelligent tire system correctly captures the expected increase in water lift force with longitudinal speed and water film depth (Figure 14 and Figure 15), demonstrating sensitivity to fundamental hydrodynamic trends. Second, the comparison with published optical measurements of the contact patch (adapted from Hermange [29]) shows that the hydroplaning risk evolution measured by the instrumented tire falls within the expected range defined by reference tires with different tread depths (Figure 16), despite differences in tire specifications and test conditions. Third, the comparison between the critical hydroplaning speed predicted by the proposed method and the results obtained from established analytical models (Gengenbach, Hermange, and Spitzhüttl) shows good agreement, with relative differences in the range of 5–8%. Taken together, these three validation pathways confirm that the proposed hydroplaning risk estimation methodology is reliable and robust.

## 5. Conclusions

This study developed and validated a real-time hydroplaning risk estimation methodology leveraging an intelligent tire system instrumented with a FlexiForce sensor. This work demonstrates, for the first time, a practical method for measuring the hydrodynamic lift generated in one of the tire tread grooves in real-world operation conditions. An analytical water intake flow rate model was derived based on the lift force model developed by Horne, providing a robust estimate of the amount of water that the tire needs to evacuate. The hydroplaning risk index is then obtained by computing the ratio between the estimated intake flow rate and the choke flow rate limit of the tire. In contrast to most existing hydroplaning studies, which focus on deep water conditions and rely on the formation of a water wedge in front of the contact patch, the method developed in this work is not dependent on this mechanism. Due to the position of the sensor on the exterior of the tire carcass, the intelligent tire is also able to estimate the hydroplaning risk in shallow water conditions where the water wedge effect is not always present. Furthermore, the direct measurement of the water lift force provides physically meaningful information about the hydroplaning mechanism. This represents an advantage compared to methods that rely on indirect measurements to estimate the risk of hydroplaning.

The proposed methodology has been applied to two data sets obtained from real-world rolling conditions at 45 mph and 65 mph, which resulted in mean hydroplaning risk values of 12.6% and 21.3%. These values are well below the 100% threshold for total hydroplaning and are consistent with previous empirical predictions, which indicated that the same tire would not reach critical conditions until approximately 190 mph (305.8 km/h) for a 0.3 mm water film depth. Frequency-domain analysis confirmed strong signal-to-noise ratios (14.6 dB at 45 mph (72.4 km/h) and 18.2 dB at 65 mph (104.6 km/h)), ensuring reliable measurement of the lift force signal even in real-world conditions.

Wet rolling tests were conducted under controlled water depth conditions of 1.05 mm at the Michelin Laurens Proving Grounds in South Carolina. Validation was performed by comparing the hydroplaning risk estimated by the intelligent tire system with data obtained from the literature through an optical imaging method. Despite differences in tire construction, loading, and test surfaces, the hydroplaning risk predicted by the intelligent tire showed close agreement with the optical imaging results reported by Hermange. Specifically, the intelligent tire, with a tread depth of 4.4 mm, exhibited hydroplaning risk levels between those observed for reference tires with 7 mm and 3 mm tread depths. Furthermore, the critical hydroplaning speed estimated by the intelligent tire system was 111 mph (178.6 km/h), falling within 5–8% of the predictions made using analytical models proposed by Gengenbach, Hermange, and Spitzhüttl.

Beyond its scientific contributions, the proposed algorithm offers a data-driven approach for estimating hydroplaning risk that can be embedded in future intelligent tire designs to enhance advanced driver-assistance systems (ADASs). A real-time hydroplaning risk signal could issue visual warnings and also inform the stability management system, giving drivers and automated vehicles additional reaction time before grip is compromised.

Future work should broaden the test matrix to include a wider range of water film depths, varied tread patterns, and the effects of inflation pressure and vertical load. For commercial implementation, future research should also assess long-term sensor durability and calibration drift. Additionally, recent advances in machine learning techniques could be coupled with the analytical flow model to improve accuracy across a wider operating envelope.

## Figures and Tables

**Table 1:** Parameters used to estimate the hydroplaning risk.

Parameter	Measurement Unit	Value
*C_L,S_*	-	0.7
*ρ*	kg/m^3^	1000
*A_S_*	m^2^	1.13 × 10^−5^
*GD*	m	0.004
*GW*	m	0.012
*p*	kPa	280
*TW*	m	0.193
*CSR*	-	0.65
*TD*	m	0.0047

## Data Availability

The original contributions presented in this study are included in the article. Further inquiries can be directed to the corresponding authors.
